# The first record of the invasive mosquito species *Aedes albopictus* in Chişinӑu, Republic of Moldova, 2020

**DOI:** 10.1186/s13071-021-05060-2

**Published:** 2021-11-03

**Authors:** Tatiana Șuleșco, Galina Bușmachiu, Unchana Lange, Jonas Schmidt-Chanasit, Renke Lühken

**Affiliations:** 1 Laboratory of Entomology, Institute of Zoology, MD-2028 Chişinӑu, Republic of Moldova; 2 Department of Arbovirology, Bernhard Nocht Institute for Tropical Medicine, Bernhard-Nocht-Str. 74, 20359 Hamburg, Germany; 3 Faculty of Mathematics, Informatics and Natural Sciences, Universität Hamburg, Hamburg, Germany

**Keywords:** *Aedes albopictus*, Asian tiger mosquito, Invasive species, Entomological survey, Republic of Moldova

## Abstract

**Background:**

In Europe, *Aedes albopictus* is an important vector of chikungunya virus and *Dirofilaria* nematodes and has been involved in local autochthonous circulation of dengue and Zika viruses. Due to the ongoing spread, targeted field surveillance at potential points of entry of invasive *Aedes* mosquitoes was initiated by the Republic of Moldova in 2020 as part of the transboundary “Invasive *Aedes* Mosquitoes COST-Action project.”

**Methods:**

In 2020, ovitraps were positioned at each of three locations: the border crossing to Romania in Leuşeni (Hancesti region), Chişinӑu International Airport and Chişinӑu Botanical Garden.

**Results:**

A total of 188 *Aedes* spp. eggs were collected at the Chişinӑu International Airport between August and September 2020. Twenty-three adults reared in the laboratory were identified morphologically as *Ae. albopictus* (Skuse, 1895), and 12 selected specimens were confirmed by molecular barcoding of the cytochrome oxidase subunit I gene region. In addition, one adult *Ae. albopictus* female at the same site was caught with a manual aspirator.

**Conclusions:**

This is the first documented report of *Ae. albopictus* in the Republic of Moldova. The presence of immature and adult stages indicates the local reproduction of the species in the country. Therefore, it is crucial to extend and strengthen surveillance of the invasive *Aedes* mosquitoes to prevent *Ae. albopictus* and other exotic mosquito species from becoming established in the Republic of Moldova.

**Graphical abstract:**

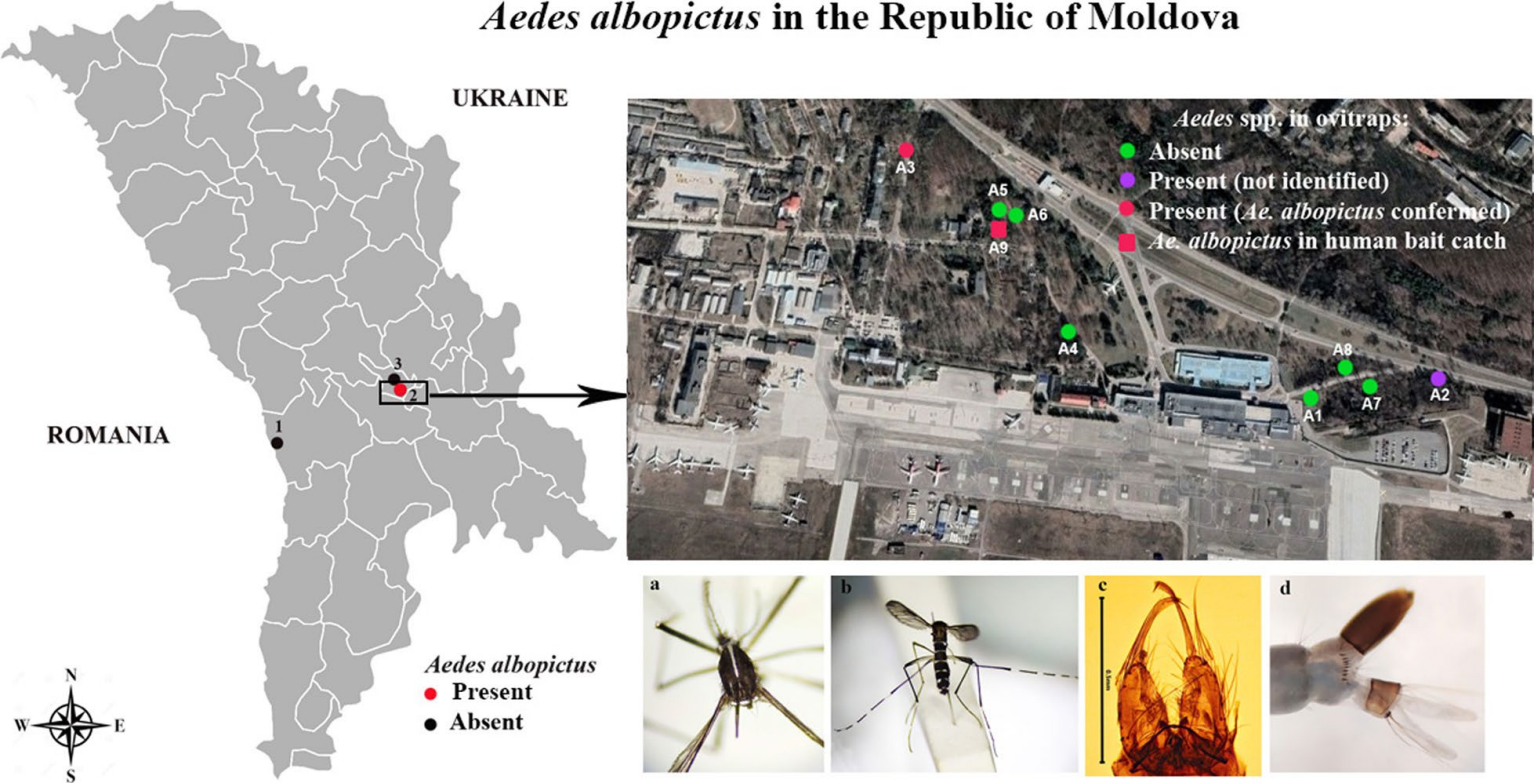

*Aedes albopictus* (Skuse, 1895), commonly known as Asian tiger mosquito, is an invasive mosquito species native to tropical and subtropical regions of Southeast Asia and the Indian Ocean [1]. Over the last 4 decades, the species rapidly expanded its distributional range worldwide including in the Americas, Africa, Australia and Europe [2]. Experimental and field data demonstrated that *Ae. albopictus* is a potential vector of > 30 different pathogens [1, 3, 4]. In Europe, *Ae. albopictus* is an important vector of chikungunya virus with several outbreaks having occurred in Italy [5, 6] and France [7–9] and *Dirofilaria* nematodes [10]. In addition, the species was involved in local autochthonous circulation of dengue virus [11–13] and Zika virus [14] in Europe.

Globalization, with increasing international trade and travel, facilitates the spread of *Ae. albopictus*. Due to its ecological plasticity [1], *Ae. albopictus* has invaded and become established in 30 countries in Europe including the neighboring regions of the Republic of Moldova in the Mediterranean Basin, the Thrace region of Turkey and the eastern Black Sea coast [15–17]. Recently, the species was introduced to the the northern Black Sea coast of the Crimean Peninsula [18]. The first report of *Ae. albopictus* in Romania, a neighboring country to the Republic of Moldova, was in Bucharest in 2012 [19]. Further sampling efforts demonstrated the spread of *Ae. albopictus* in the country, including the Constanta region, where positive sampling sites were close to the border of Moldova [20].

The global trade of lucky bamboo (*Dracaena* sp.) and tires is the most important pathway for the global dispersal of invasive *Aedes* species [21, 22]. Subsequently, public and private transport especially along highways is considered one of the main drivers of *Ae. albopictus*'s spread in Europe [23–27]. Thus, targeted field surveillance of potential points of entry (PoE) for *Ae. albopictus* and other invasive *Aedes* mosquitoes (AIM) at a highway, botanical garden and airport was carried out in 2020 in the Republic of Moldova as part of the “AIM-COST action project” [28].

Field surveys were conducted at three locations that were potential routes of entry to the Republic of Moldova for invasive *Aedes* mosquito species. These included the border crossing to Romania in Leuşeni, Hânceşti region (10 June–16 October 2020), where the border inspection post is located in an agricultural environment and international vehicular transport regularly enters the country, Chişinӑu International Airport (5 July–31 October 2020) and Chişinӑu Botanical Garden (10 July–16 October 2020), known for introducing and growing tropical plants (Fig. 1). Conical-shaped black plastic containers (height: 13 cm, lower diameter: 9 cm, upper diameter: 13 cm) with approximately 1-l volume were used as ovitraps [15]. Each trap was filled two thirds with clean water, and scratched tongue depressors (1.7 × 15 cm) were added as an egg-laying substrate for invasive *Aedes* species. Five ovitraps were positioned in each location in the shaded sites at a distance not less than 20 m from each other, and the maximum distance between traps was about 400 m. (Fig. 1). The tongue depressors and water were collected from the traps and replaced with clean water and new tongue depressors every 2 weeks. All samples were transported to the Entomology Laboratory, Institute of Zoology, in Chişinӑu for rearing of eggs and larvae to adults in trays containing dechlorinated water. Hatched larvae were fed with aquarium fish food (ASTRA Aquaristik GmbH, Osnabrück, Germany). Morphological species identification of larvae and adults was conducted with the keys in Becker et al. [29].

**Fig. 1:**
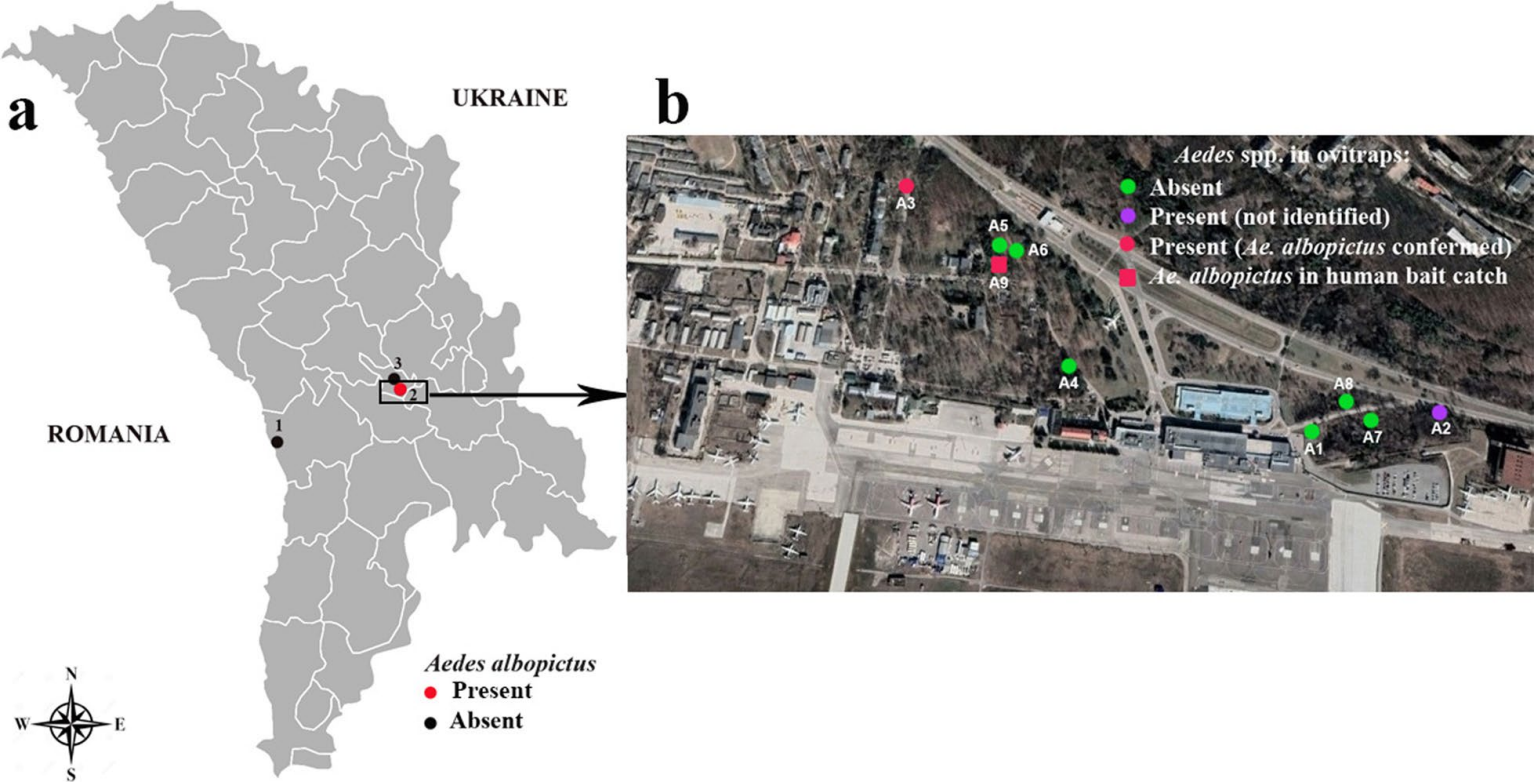
Study area. **a** Sampling locations in the Republic of Moldova: 1: the border crossing to Romania in Leuşeni; 2: Chişinӑu International Airport; 3: Chişinӑu Botanical Garden. **b** Sampling sites at Chişinӑu International Airport

Two mosquito taxa were collected from the ovitraps during the entomological surveys: *Culex pipiens* (sensu lata)/*Cx. torrentium* (205 individuals) and *Aedes* spp. (188 eggs) (Table 1). *Culex pipiens* (s.l.)/*Cx. torrentium* was present at all three study locations, while *Aedes* spp. eggs were only collected at Chişinӑu International Airport. Two ovitraps positioned in the forest square close to the airport collected 188 *Aedes* spp. eggs. The first positive ovitrap (A3: latitude 46.938, longitude 28.928, altitude 80 m) yielded 167 eggs: 72 eggs (21 August), 38 eggs (5 September) and 57 eggs (27 September). The second trap (A2: latitude 46.936, longitude 28.940, altitude 80 m) collected 21 *Aedes* spp. eggs on 27 September. Twenty-three specimens (19 females and 4 males) were successfully reared from the *Aedes* spp. eggs to adult stage and identified as *Ae. albopictus* by larval and adult morphology (Fig. 2). Morphological identification of *Ae. albopictus* was confirmed by molecular barcoding of the cytochrome oxidase subunit I gene region of 12 randomly selected specimens [30]. All sequences were entered into GenBank (accession no. MZ069031–MZ069042). In addition, one *Ae. albopictus* female was caught by manual aspirator during ovitrap inspection at the airport on 27 September (Fig. 1b). Three additional ovitraps (A6, A7, A8) were placed at the Chişinӑu International Airport at the end of September and surveillance continued through to 31 October, but no further *Aedes* spp. eggs were collected.

**Table 1:** *Aedes* spp. eggs with confirmed *Ae. albopictus* specimens and *Cx. pipiens* (s.l.)/*Cx. torrentium* larvae collected from 15 ovitraps at three sampling locations in the Republic of Moldova, 2020

Location	*Aedes* spp. eggs	Hatched *Aedes* spp. (confirmed as *Ae. albopictus* by morphology/tested and confirmed by COI barcoding)	*Culex pipiens* (s.l.)/*Cx. torrentium*
Airport, Chişinӑu			
A1			59
A2	21	0	
A3	167	23 (23/12)	
Botanical garden, Chişinӑu			
B1			28
B2			20
Leuşeni, Hânceşti			
C1			15
C2			83
Total	188	23	205

**Fig. 2:**
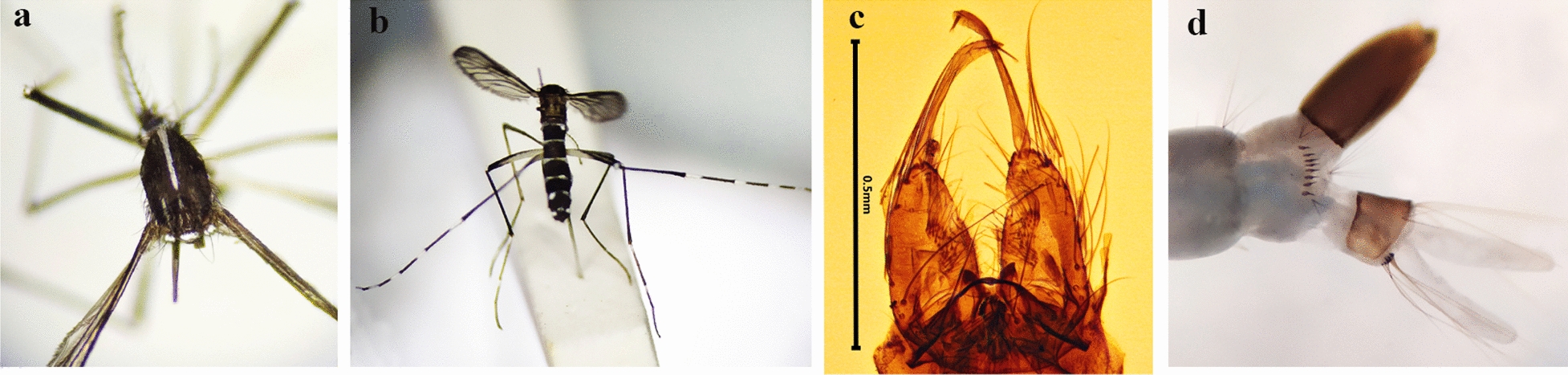
Specimens of *Aedes albopictus* collected at the Chişinӑu International Airport in 2020. **a** Adult female, pattern on scutum; **b** adult female, dorsal view, **c** male genitalia; **d** fourth-instar larva

Surveillance of the presence/absence of invasive *Aedes* species at the potential PoE in the Republic of Moldova demonstrated the presence of *Ae. albopictus* at the Chişinӑu International Airport. In the past, only few studies have been dedicated to the role of European airports in importing exotic mosquito species with *Ae. albopictus* recorded at Schiphol Airport, The Netherlands [31–34].

The introduction of *Ae. albopictus* in Europe was facilitated by passive dispersion through the global transportation of tires [35, 36] and the import of *Dracaena* plants known as “lucky bamboo,” e.g., in The Netherlands [21] and Bulgaria [37]. Further dispersal in Europe inside vehicles via highway systems was documented in Switzerland [38], Germany [23], Spain [24] and the UK [26]. However, no exotic mosquito species were detected at the border crossing between Romania and Chişinӑu and at the botanical garden.

This study emphasizes the importance of air transportation for the dispersal of *Ae. albopictus* in Europe. This is the first documented report of *Ae. albopictus* in the Republic of Moldova to our knowledge, and the presence of adult and immature stages indicates the local reproduction of the species. Further investigations with greater trapping efforts are necessary to clarify whether this is a stable, established population. This is especially important for determining future mosquito control measures; public health authorities were informed, but insecticidal control has not yet been implemented. In addition, with the increasing spread and population densities of *Ae. albopictus* in Europe, additional introductions have to be expected via air traffic and other routes of entry, which may allow long-term establishment. Therefore, it is crucial to extend and strengthen surveillance of invasive *Aedes* mosquitoes to prevent their establishment and future arbovirus transmission in the Republic of Moldova.

## Data Availability

All data generated or analyzed during this study are included in this published article.

## Abbreviations

PoE: Points of entry
AIM: *Aedes* invasive mosquitoes
